# The chemical composition and toxic effects of aqueous extracts of *Cyclocarya paliurus* leaves

**DOI:** 10.3389/fnut.2022.994055

**Published:** 2022-09-29

**Authors:** Cencen Liu, Mouming Zhao, Lingrong Wen, Hongwei Zhao

**Affiliations:** ^1^School of Food Science and Engineering, South China University of Technology, Guangzhou, China; ^2^Infinitus (China) Company Ltd., Guangzhou, China; ^3^South China Botanical Garden, Chinese Academy of Sciences, Guangzhou, China

**Keywords:** safety, *Cyclocarya paliurus* leaves, MTD, chemical composition, toxicity

## Abstract

*Cyclocarya paliurus* leaves, which possess various bioactivities, have been widely used in dietary supplements or as ingredients in functional foods. However, limited information is available about the toxicity or safety concerns. In the present work, the maximum tolerated dose (MTD) and potential toxicity of the aqueous extracts of *C. paliurus* leaves (AECPL) were evaluated. Our results indicated that AECPL was rich in phenolics, flavonoids, and polysaccharides, which might be responsible for the health benefits of *C. paliurus* leaves. The MTD of AECPL was considered to be > 10,000 mg/kg BW in both male and female rats. The acute toxicity study was carried out by a 14-day repeat dose oral toxicity study. The results showed that the rats were all well-tolerated. No treatment-related mortality, abnormal clinical signs, body weight, or food consumption changes were reported during the study. Moreover, AECPL showed no adverse changes in the hematology, serum chemistry, urinalysis parameters, organ weights, gross finding, and histopathology. In this study, the non-observed-adverse-effect level of AECPL was 5,000 mg/kg BW/day, indicating AECPL was safe and can be used in the food industry.

## Introduction

*Cyclocarya paliurus* (Batalin) Iljinskaja (*C*. *paliurus*), belonging to the genus of *Cyclocarya* Iljinskaja, is a well-known edible herbal medicine that is grown in southern China (1, 2). *C*. *paliurus* is commonly named as “sweet tea tree” due to the flavor of its leaves (3). Traditionally, the leaves of *C*. *paliurus* are used for drug formulations with the curative effects of eliminating phlegm-turbid stasis and dampness-heat obstruction in traditional Chinese medicine (4). Recently, they have also been used in folk medicine for the treatment of anti-hypertensive, diabetes, hypertension, and hyperlipoidemia (5). Moreover, *C. paliurus* leaves have been widely used as dietary supplements or ingredients in functional foods in China. A series of *C*. *paliurus* leaf-derived products, for example, teas, beverages, and so on, have been developed (6). It is worth noting that *C. paliurus* leaves are the first officially approved health tea in China since 1999 (7).

To date, a larger number of bioactive components, including flavonoids (such as quercetin, isoquercitrin, quercetin-3-*O*-β-d-glucuronide, kaempferol, kaempferol-3-*O*-β-d-glucuronide, and kaempferol-7-*O*-α-l-rhamnoside), polysaccharides, triterpenoids, saponins, and steroids have been identified in *C. paliurus* leaves (1, 8, 9). Additionally, various biological activities, including cholesterol-lowering, hypoglycemic, hypolipidemic, antioxidant, antihypertensive, anti-inflammation, and immunomodulatory effects have been reported for *C*. *paliurus* leaves (10). At present, scientists have paid more attention to *C. paliurus* leaves due to the presence of abundant numerous bioactive phytochemicals and their health benefits (11). It is necessary to ensure the oral safety of *C. paliurus* leaves. However, limited information is available about the acute toxicity of extracts from *C. paliurus* leaves. Therefore, an oral dose toxicity study of *C. paliurus* leaves extract in rats, including male and female, was carried out in the present work. Moreover, the chemical compositions of *C. paliurus* leaves extract were investigated as well. The results may help to provide a security basis and quality standard of *C. paliurus* leaves as a functional food ingredient with various bioactivities.

## Materials and methods

### Plant materials and chemicals

Dried leaves of *C. paliurus*, provided by Guangdong Tiansheng Pharmaceutical Co., Ltd. (Foshan, China), were milled into powder to obtain a fine powder of about 60 mesh, and were stored under –20°C until used. Gallic acid, Folin-Ciocalteu reagent, and rutin were purchased from Sigma Chemical Co. (St. Louis, MO, United States). Sodium carbonate (NaCO_3_), aluminum nitrate (AlNO_3_), sodium nitrite (NaNO_2_), sodium hydroxide (NaOH), and ethanol were obtained from Guangzhou Reagent Co. (Guangzhou, China).

### Tested animals

Rats (*Rattus norvegicus*)/Crl:CD^®^ [SD] VAF/Plus^®^ (5–6 weeks of age), including male and female, were purchased from Vital River Laboratory Animal Technology Co., Ltd. (Beijing, China). Before treatment, all animals were quarantined/acclimated per facility SOPs. The rats were group housed (up to three animals of the same sex and same dosing group together) in solid bottom cages. All the animals were kept under standard conditions (temperature, 22 ± 2°C; humidity, 40–70%; 12-h light/dark cycle), and were supplied with distilled water *ad libitum* and a standard diet (Rodents Irradiated Maintenance Feed *ad libitum*) throughout the experiment period. All the experimental protocols and/or procedures involving the animal’s test in the present work had been reviewed and approved by the WuXi AppTec Institutional Animal Care and Use Committee before the initiation of such procedures.

### Extraction

The bioactive components from *C. paliurus* leaves were extracted by hot water according to the previous report (6) with some modifications. In brief, the milled leaves were mixed with distilled water at a ratio of 1:10 (w/v), then the mixture was extracted at 100°C for 60 min. After centrifugation (5,000 × *g*, 10 min), the supernatants were collected, and the residues were further extracted with distilled water at 100°C for 40 min. All the supernatants were combined and concentrated to a concentration equivalent to 1 g/ml (leaves/water) under vacuum at 90°C. The aqueous extracts of *C. paliurus* leaves (AECPL) were obtained after free-drying and were stored at –20°C until use.

### Chemical compositions determination

The compositions of AECPL were determined in this study. For the content of polysaccharides, AECPL was dissolved in 80% ethanol to remove ethanol-soluble chemicals, then the residues were dissolved in distilled water and the polysaccharides content of AECPL was measured by the phenol-sulfuric acid method as described previously (12). The final result was expressed as gram glucose equivalents (GE) per 100 grams of AECPL (g GE/100 g AECPL). The protein content of AECPL was measured by automatic Kjeldahl assay according to a previous report (13), and the final result was expressed as gram per 100 grams of AECPL (g/100 g AECPL). The total phenolic content of AECPL was determined by Folin–Ciocalteu colorimetric method as described previously (14), using gallic acid as the standard. The final result was expressed as a gram of gallic acid equivalents (GAE) per 100 grams of AECPL (g GAE/100 g AECPL). The total flavonoid content of AECPL was measured by aluminum chloride colorimetric assay as described by Wen et al. (14), using rutin as the standard. The final results were expressed as a gram of rutin equivalents (RE) per 100 grams of AECPL (g RE/100 g AECPL).

UHPLC-MS-MS analysis was applied for the phytochemicals profile of AECPL by using an UltiMate 3000 UHPLC DGLC Systems equipped with a Q EXACTIVE QE mass spectrometer (ThermoFisher Scientific) and a Phenomenex C18 column (4.6 × 250 mm, 5.0 μm, Phenomenex, United States). The samples were eluted with a gradient system consisting of solvent A (H_2_O with 0.1% formic acid, v/v) and solvent B (acetonitrile), which were used as the mobile phase, with a flow rate of 0.4 ml/min. The column temperature was kept at 35°C and the injection volume was 1 μl. Gradient elution program was applied for analysis and set as follows: 0–10 min with 3–9% solvent B, 10–12 min with 9–10% B, 12–16 min with 10–13% B, 16–30 min with 13–19% B, 30–60 min with 19–35% B, 60–70 min with 35–97% B, 70–79 min with 97% B, 79–80 min with 97-3%B, and kept 3% B for 8 min. MS analysis with an electrospray ionization (ESI) source was operated in a negative ion model. MS data were collected in a full-scan model with a mass range of 100–1,500 Da. The capillary and heater temperatures were 350°C. The sheath gas flow rate and aux gas flow rate were 40 and 10 L/h, respectively. The cone voltage was 20 kV. The tentative identification and structural characterization of major compounds were based on MS and MS/MS spectra and were confirmed by the previous report.

### Animals’ treatment

**FIGURE 1:**
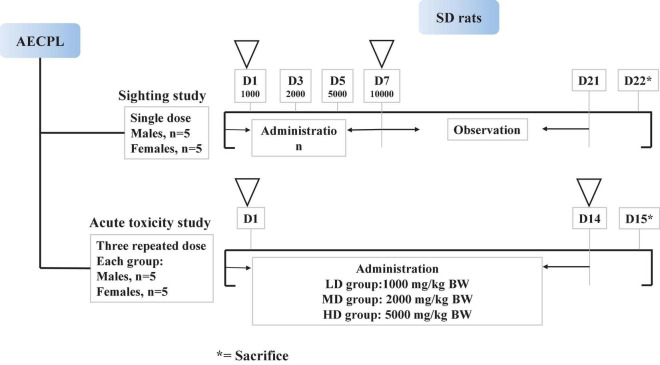
Scheme of animals’ treatment.

A sighting study was performed to determine the maximum tolerated dose (MTD) of AECPL through oral gavage administration (15). After acclimation for 6 days before experimentation, 10 rats (5 males and 5 females) were randomly assigned to two groups (group A: 5 males and group B: 5 females). As shown in Figure 1, all the tested animals received AECPL in distilled water at ascending doses (1,000, 2,000, 5,000, or 10,000 mg/kg BW for once) at approximately 48 h intervals through oral gavage administration. Then, the rats were observed for 14 consecutive days before the final sacrifice, following the last dose (Figure 1). The mortality and clinical observations of the tested animals were recorded for 21 days. On the 22nd day, rats were dissected and evaluated for macroscopic lesions after sacrificing by cervical dislocation.

The acute toxicity study was carried out by a 2-week repeated oral dose toxicity study. After acclimation for 6 days before experiment, 40 rats (20 males and 20 females) were randomly assigned to 4 groups, which included 5 each of males and females. The tested animals were administered by oral gavage AECPL in the doses of 0 (NC group, normal control), 1,000 (LD group, low-dose), 2,000 (MD group, middle-dose), and 5,000 mg/kg BW/day (HD group, high-dose), respectively, for 14 consecutive days. The rats were euthanatized a day after the last dose. Rats with AECPL treatment received the same volume of AECPL solution at the above-mentioned dose, while the animals in the NC group received the same volume of vehicle solution (distilled water). The mortality and clinical observations of the tested animals were recorded for 15 days.

### Clinical observations and body weight measurement

For both the sighting study and repeated oral toxicity study, all the tested animals were observed two times daily for mortality and morbidity. Clinical signs, including abnormal behavioral and physical changes, as well as changes in the fur, skin, eyes, breathing, and excretion were recorded. For a single dose toxicity study, the body weights were measured on 1, 3, 5, 7, 10, 14, 17, and 21-day. For repeated oral dose toxicity study, the body weights were measured on 1, 4, 8, 11, and 14-day.

### Clinical pathology

At the end of the experiment period (day 15), all the tested animals in the repeated oral dose toxicity study were anesthetized with isoflurane, and the blood samples were collected from the abdominal aorta and centrifuged immediately for 5 min at 14,000 *g* at 4°C to obtain serum for biochemical analysis. In addition, the whole blood samples were collected using sodium citrate as the anticoagulant. Urine was collected overnight by placing the rats in metabolic cages where animals had access to water without food. After the collection of blood and urine samples, hematology, clinical chemistry, and urinalysis were performed. The tested rats were fasted overnight before blood/urine collection.

### Histopathology

A detailed gross necropsy, including a comprehensive gross observation of external surfaces of the body, orifices, cranial, thoracic, and abdominal cavity were conducted on all tested animals in both sighting and repeated oral dose toxicity studies. The adrenal glands (AG), brain, heart, kidneys, liver, ovaries/testes, spleen, and thymus of all animals were trimmed of any adherent tissues. Their wet weight was recorded. Tissues or organs were fixed in 10% neutral buffered formalin or other appropriate fixative and then prepared for histopathological examination from the NC group and HD group animals.

### Statistical analyses

All data were expressed as means ± standard deviations of each measurement. Statistical analyses were carried out in male and female groups, respectively. The homogeneity of the group variances was evaluated using Levene’s test at the 0.05 significance level. If differences between group variances were not significant (*p* > 0.05), then a parametric one-way analysis of variance (ANOVA) was performed. When significant differences among the means were indicated by the ANOVA test (*p* ≤ 0.05), Dunnett’s test was used to perform the group mean comparisons between the control group and each treated group.

## Results and discussion

### Chemical compositions of aqueous extracts of *Cyclocarya paliurus* leaves

The chemical compositions of AECPL, including polysaccharides, total phenolics, total flavonoids, protein, and quercetin, contents were determined. Our results indicated that the polysaccharides content was 15.84 ± 1.54 g GE/100 g AECPL. The total phenolic content was 28.15 ± 1.95 g GAE/100 g AECPL. The total flavonoid content was 16.99 ± 0.83 g RE/100 g AECPL. Additionally, the content of protein was 3.35 ± 0.34 g/100 g AECPL. UHPLC-MS/MS, which has been proven to be a sensitive method in detecting and characterizing trace constituents (14), was used to distinguish phytochemicals in AECPL. As shown in Table 1, 15 phenolic compounds, including 6 phenolic acids (protocatechuic acid, 4-dicaffeoylquinic acid, chlorogenic acid, neochlorogenic acid, caffeic acid, and 4,5-di-*O*-caffeoylquinic acid) and 9 flavonoids were identified. Among the nine flavonoids, seven were flavonoid-*O*-glycosides, which included quercetin-*O*-galactoside, isoquercitrin, quercetin-*O*-glucuronide, kaempferol-*O*-glucuronide, kaempferol-*O*-glucoside, quercetin-*O*-rhamnoside, and kaempferol-*O*-rhamnoside; two were flavonols including kaempferol and quercetin.

**TABLE 1:** The identified phenolics compounds of aqueous extracts of *Cyclocarya paliurus* leaves based on the UHPLC-ESI-MS/MS analysis.

No.	RT (min)	Identification	[M-H]^–^ (m/z)	MS^2^/(m/z)
1	14.90	Protocatechuic acid	153.0184	109.0284
2	17.12	4-Dicaffeoylquinic acid	353.0877	191.0554, 179.0342, 135.0441
3	22.06	Chlorogenic acid	353.0876	191.0554, 173.0447
4	23.14	Neochlorogenic acid	353.0876	191.0554, 179.0342, 173.047, 135.0441
5	24.70	Caffeic acid	179.0341	135.0441
6	38.25	Quercetin-*O*-galactoside	463.0879	301.0353
7	38.84	Isoquercitrin	463.0881	301.035
8	41.11	Quercetin-*O*-glucuronide	477.0672	301.0353, 178.9979, 151.0029, 145.0285
9	44.89	Kaempferol-*O*-glucuronide	461.0725	285.0403
10	43.08	Kaempferol-*O*-glucoside	447.0932	285.0403
11	43.38	Quercetin-*O*-rhamnoside	447.0929	301.0353
12	45.55	4,5-di-O-Caffeoylquinic acid	515.1191	353.0876, 173.0448
13	48.38	Kaempferol-*O*-rhamnoside	431.0978	285.0402
14	64.37	Kaempferol	285.0403	151.0027
15	72.78	Quercetin	301.0775	151.0026

Different types of chemical compounds, including proteins, polysaccharides, phenolics, and triterpenoids were observed in *C*. *paliurus* (16). Among them, polysaccharides are one of the major bioactive components presented in *C*. *paliurus* leaves (1, 5). A previous report reviewed that the monosaccharides, such as rhamnose, arabinose, galactose, glucose, mannose, galacturonic acid, xylose, and glucuronic acid were found in the polysaccharides from *C*. *paliurus* leaves. Among these monosaccharides, rhamnose, arabinose, galactose, and galacturonic acid were the most dominant ones, implying the presence of pectin-like polysaccharides in *C*. *paliurus* leaves (11). Moreover, a novel pectin-like polysaccharide with high water solubility was purified from the dried leaves of *C*. *paliurus* (17). In addition, polysaccharides from *C*. *paliurus* showed various biological activities, which included anticancer, antioxidant, antimicrobial, antidiabetic, and anti-hyperlipidemic activities, as well as immunomodulatory effects (11). Phenolic acids and flavonoids are the major phenolic compounds present in *C*. *paliurus* leaves. A chemical fingerprint analysis by Cao et al. (18) showed that three phenolic acids (4,5-di-*O*-caffeoylquinic acid, and esters of caffeic acid and quinic acid) and eight flavonoids (quercetin-3-glucuronide, quercetin-3-galactoside, isoquercitrin, quercetin-3-rhamnoside, kaempferol-3-glucuronide, kaempferol-3-rhamnoside, and kaempferol-3-glucoside) were identified in *C*. *paliurus* leaves. These phenolic acids and flavonoids were considered to be the marker compounds of *C*. *paliurus* leaves. Our results indicated that phenolic acid and flavonoids were the major phytochemicals in *C*. *paliurus* leaves, which is in accordance with a previous study, in which quercetin-3-glucuronide, kaempferol-3-glucuronide, kaempferol-7-rhamnoside, and kaempferol were found to be the main flavonoids in *C*. *paliurus* leaves (8). *C*. *paliurus* extract rich in quercetin, kaempferol-3-glucuronide, isoquercitrin, and kaempferol-3-rhamnopyranoside could ameliorate the imbalanced intestinal microbiota induced by circadian rhythm disorder (19). Previous study also indicated that total flavonoids from *C*. *paliurus* leaves showed hepatoprotective effect against carbon tetrachloride-induced acute liver injury *in vivo* (20). All these results indicated that the polysaccharides, phenolics, and flavonoids presenting in *C*. *paliurus* leaves extract might contribute to their bioactivities.

### The maximum tolerated dose of aqueous extracts of *Cyclocarya paliurus* leaves

**FIGURE 2:**
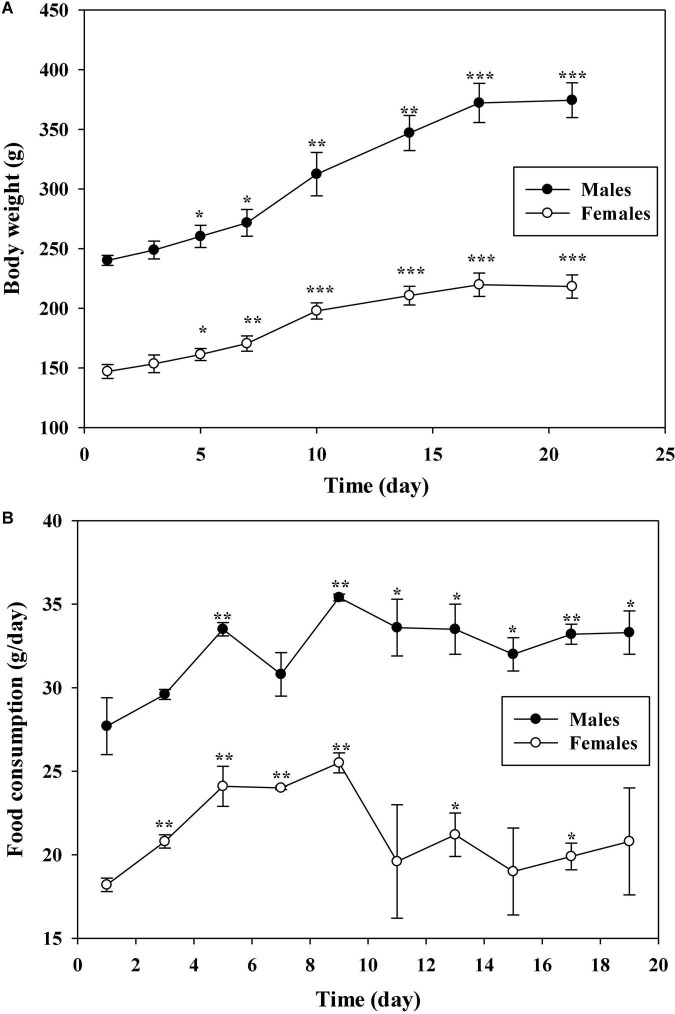
The effects of AECPL on the body weights **(A)** and food consumption **(B)** of Sprague-Dawley (SD) rats during the sighting study. “*, **, and ***” refers to a significant difference from the 1st day at *p* ≤ 0.05, 0.01, and 0.001, respectively.

The MTD, a dose in which there is no mortality of animals after administration with the tested samples, was determined using a sighting study in both males and females Sprague-Dawley (SD) rats. Dosing of AECPL started at 1,000 mg/kg BW in rats through oral administration, and sequential higher doses of 2,000, 5,000, and 10,000 mg/kg BW were administered to animals, and no sign of mortality was observed within 7 days. Then, the animals were observed for 14 consecutive days. No associated mortality and adverse clinical symptoms were observed within 14 days of post-administration. The body weight and feed intake of tested animals were recorded as well. As shown in Figure 2A, the body weight was increased in a time-of-day-dependent manner from day 1 to day 17 after administration, in both male and female groups, and remained constant from day 17. Similarly, the feed intake was increased from day 1 to day 9, and remain constant from day 11 (Figure 2B). All the changes were in accordance with those of other unadministered rats in the lab (data not shown), suggesting that no adverse effects on the body weight and feed intake were caused by AECPL. Additionally, no sighting lesions were found in the tested rats at necropsy. All these results indicated that AECPL was well-tolerated in SD rats when administered once by oral gavage at doses varied from 1,000 to 10,000 mg/kg BW. Therefore, the MTD of AECPL was considered to be more than 10,000 mg/kg BW in both male and female rats. Similarly, Deng et al. (6) found that AECPL was well-tolerated in Kunming mice after oral gavage at a dose of 10,000 mg/kg BW. Moreover, the genotoxicity and teratogenicity study of AECPL indicated that administration with AECPL at 10,000 mg/kg BW showed non-mutagenic and non-teratogenic effects in SD rats, suggesting the safety of AECPL at 10,000 mg/kg BW.

### Effects of aqueous extracts of *Cyclocarya paliurus* leaves on the body weights and feed intake of Sprague-Dawley rats in repeated oral dose toxicity study

**FIGURE 3:**
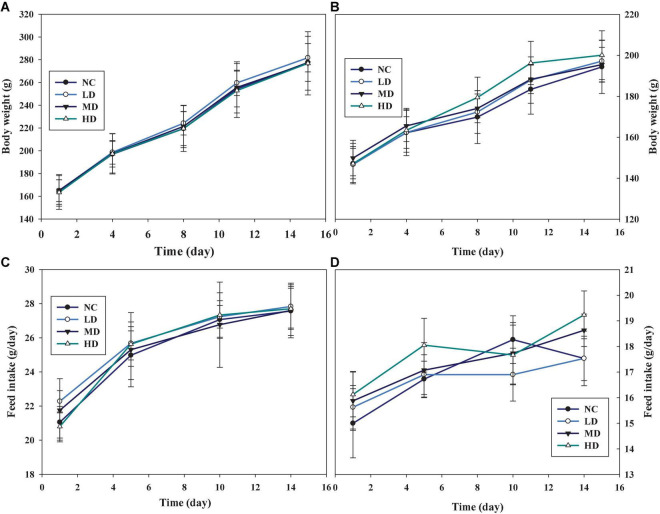
The effects of AECPL on the body weights and feed intake on Sprague-Dawley (SD) rats in acute toxicity study. **(A,B)** refer to the body weights of males and females, respectively; **(C,D)** refer to the feed intake of males and females, respectively.

Considering the results of the sighting study, a repeated oral dose toxicity study was performed at a series of doses of 1,000, 2,000, and 5,000 mg/kg BW for 14 days. Similar trends of body weight, which increased gradually were found in the groups administrated with AECPL, in both male (Figure 3A) and female (Figure 3B) rats. And the body weights of the treated rats were not significantly different (*p* ≥ 0.05) as compared to the untreated rats. Similar results were observed for food intake (Figures 3C,D). Additionally, no associated mortality and adverse clinical symptoms were observed during this study. All these results suggested that administration with repeated doses of AECPL did not cause observable toxicity in SD rats. A previous study mentioned that the ethanol extract of the stems and leaves of *C*. *paliurus* did not cause any mortalities or body weight changes at the doses of 1,000 and 2,000 mg/kg BW in male Swiss Webster mice (21). However, the raw data for the acute toxicity study were not provided in the reports.

### Effects of aqueous extracts of *Cyclocarya paliurus* leaves on the clinical pathology of Sprague-Dawley rats in repeated oral dose toxicity study

Post dosing period, the clinical pathology, including hematology, serum biochemistry, and urinalysis, parameters were measured. And the results are presented in Tables 2–4. As shown in Table 2, leukocyte counts (WBC), erythrocyte count (RBC), hemoglobin (HGB), hematocrit (HCT), mean corpuscular volume (MCV), mean corpuscular hemoglobin (MCH), mean corpuscular hemoglobin concentration (MCHC), RBC distribution width (RDW), platelet count (PLT), mean platelet volume (MPV), reticulocyte count (RET), and the absolute values and percent of different WBC classification, including neutrophils (NEUT), eosinophils (EOS), basophils (BASO), monocytes (MONO), and lymphocytes (LYMP), in the blood samples were determined. Compared with the NC group, no significant differences (*p* ≥ 0.05) were observed for all the parameters in male rats, regardless of the tested doses. Whereas, in females, the RET and EOS in the LD group were significantly different (*p* < 0.05) from the NC group, and no significant differences (*p* ≥ 0.05) were found in other parameters.

**TABLE 2:** Effects of aqueous extracts of *Cyclocarya paliurus* leaves on the hematology of Sprague-Dawley (SD) rats in the acute toxicity study.

Haemogram	WBC (10^3^/μl)	RBC (10^6^/μl)	HGB (g/dL)	HCT (%)	MCV (fL)	MCH (pg)	MCHC (g/dL)	RDW (%)	RET (10^9^/L)	NEUT (10^3^/μl)	%NEUT (%)
Males											
NC	8.6 ± 2.7	7.0 ± 0.3	14.1 ± 0.5	42.4 ± 1.6	60.4 ± 1.1	20.1 ± 0.6	33.3 ± 0.4	12.6 ± 0.2	320.0 ± 10.5	0.7 ± 0.1	8.5 ± 2.1
LD	8.6 ± 1.6	6.8 ± 0.3	14.0 ± 0.4	42.4 ± 1.0	62.4 ± 2.0	20.6 ± 0.5	33.0 ± 0.4	12.8 ± 0.3	345.8 ± 19.6	0.7 ± 0.3	8.1 ± 2.8
MD	10.2 ± 3.7	6.8 ± 0.4	13.8 ± 0.6	42.0 ± 1.8	61.4 ± 1.8	20.2 ± 0.6	32.8 ± 0.2	13.2 ± 0.7	354.8 ± 53.7	0.7 ± 0.3	7.1 ± 2.0
HD	9.1 ± 2.4	7.0 ± 0.2	13.7 ± 0.2	41.6 ± 0.5	59.3 ± 1.5	19.5 ± 0.5	32.8 ± 0.2	12.6 ± 0.3	354.0 ± 39.1	0.8 ± 0.4	8.6 ± 2.3
Females											
NC	6.5 ± 2.5	7.2 ± 0.2	13.8 ± 0.5	41.2 ± 1.4	57.6 ± 2.0	19.3 ± 0.6	33.6 ± 0.5	11.8 ± 0.4	256.4 ± 17.6	0.6 ± 0.3	9.6 ± 3.7
LD	7.1 ± 1.4	7.3 ± 0.4	13.8 ± 0.5	40.6 ± 1.8	56.0 ± 0.5	19.1 ± 0.4	34.0 ± 0.4	11.9 ± 0.2	204.2 ± 27.4**	0.5 ± 0.1	7.1 ± 2.5
MD	7.4 ± 2.2	7.1 ± 0.3	13.8 ± 0.7	40.7 ± 1.7	57.1 ± 0.5	19.4 ± 0.3	34.0 ± 0.4	11.7 ± 0.2	219.4 ± 18.9	0.6 ± 0.3	8.0 ± 2.8
HD	6.5 ± 1.4	6.9 ± 0.3	13.6 ± 0.6	40.0 ± 1.4	58.0 ± 1.6	19.8 ± 0.7	34.0 ± 0.3	11.8 ± 0.1	225.9 ± 25.2	0.5 ± 0.1	7.8 ± 2.0

**Groups**	**LYMP** **(10^3^/μl)**	**%LYMP** **(%)**	**MONO** **(10^3^/μl)**	**%MONO** **(%)**	**EOS** **(10^3^/μl)**	**%EOS** **(%)**	**BASO** **(10^3^/μl)**	**%BASO** **(%)**	**PLT** **(10^3^/μl)**	**MPV** **(fL)**

Males										
NC	7.6 ± 2.6	87.5 ± 2.9	0.13 ± 0.02	1.6 ± 0.4	0.04 ± 0.01	0.5 ± 0.2	0.02 ± 0.01	0.2 ± 0.1	1193 ± 107	7.0 ± 0.3
LD	7.6 ± 1.4	88.4 ± 3.3	0.13 ± 0.02	1.5 ± 0.4	0.04 ± 0.02	0.5 ± 0.2	0.02 ± 0.01	0.2 ± 0.0	1146 ± 68	7.2 ± 0.7
MD	9.0 ± 3.4	87.7 ± 4.6	0.18 ± 0.11	1.8 ± 0.8	0.06 ± -0.04	0.5 ± 0.3	0.03 ± 0.01	0.3 ± 0.0	1042 ± 536	7.4 ± 0.7
HD	7.9 ± 1.8	87.6 ± 2.8	0.15 ± 0.07	1.5 ± 0.5	0.04 ± 0.03	0.4 ± 0.3	0.02 ± 0.01	0.2 ± 0.1	1127 ± 97	7.3 ± 0.4
Females										
NC	5.7 ± 2.2	86.8 ± 3.6	0.10 ± 0.06	1.4 ± 0.6	0.04 ± 0.01	0.7 ± 0.2	0.01 ± 0.01	0.2 ± 0.0	1256 ± 101	7.3 ± 0.3
LD	6.3 ± 1.3	89.1 ± 2.6	0.08 ± 0.04	1.1 ± 0.3	0.07 ± 0.02*	1.0 ± 0.3	0.01 ± 0.00	0.1 ± 0.0	1164 ± 129	7.3 ± 0.4
MD	6.6 ± 2.0	88.0 ± 3.9	0.12 ± 0.07	1.5 ± 0.7	0.06 ± 0.02	0.9 ± 0.5	0.02 ± 0.01	0.2 ± 0.1	1174 ± 61	7.0 ± 0.1
HD	5.8 ± 1.4	89.1 ± 2.4	0.06 ± 0.01	1.0 ± 0.3	0.04 ± 0.01	0.7 ± 0.2	0.01 ± 0.01	0.2 ± 0.1	1124 ± 121	7.1 ± 0.3

WBC, leukocyte count; RBC, erythrocyte count; HGB, hemoglobin; HCT, hematocrit; MCV, mean corpuscular volume; MCH, mean corpuscular hemoglobin; MCHC, mean corpuscular hemoglobin concentration; RDW, RBC distribution width; RET, reticulocyte count; NEUT, neutrophils; LYMP, lymphocytes; MONO, monocytes; EOS, eosinophils; BASO, basophils; PLT, platelet count; MPV, mean platelet volume.

“*” and “**” refer to significant difference from the NC group at *p* ≤ 0.05 and *p* ≤ 0.01, respectively.

**TABLE 3:** Effects of aqueous extracts of *Cyclocarya paliurus* leaves on the serum chemistry of Sprague-Dawley (SD) rats in the acute toxicity study.

Groups	ALT (U/L)	AST (U/L)	TP (g/L)	ALB (g/L)	TBIL (μmol/L)	ALP (U/L)	GLU (mmol/L)	Urea (mmol/L)	CRE (μmol/L)	Ca (mmol/L)
Male rats										
NC	35.0 ± 4.0	123.0 ± 13.0	52.9 ± 0.9	31.4 ± 0.6	2.2 ± 0.4	222.0 ± 55.0	8.0 ± 0.8	5.6 ± 0.5	20.0 ± 3.0	2.4 ± 0.0
LD	30.0 ± 3.0	106.0 ± 17.0	53.6 ± 1.0	31.6 ± 0.7	2.3 ± 0.4	213.0 ± 49.0	9.1 ± 1.2	4.9 ± 0.5	17.0 ± 1.0	2.4 ± 0.1
MD	31.0 ± 4.0	104.0 ± 22.0	54.5 ± 1.5	31.9 ± 0.7	2.3 ± -0.5	215.0 ± 33.0	9.4 ± 0.9	5.3 ± 1.0	18.0 ± 2.0	2.5 ± 0.0
HD	37.0 ± 3.0	112.0 ± 16.0	53.3 ± 1.6	31.6 ± 0.5	2.4 ± 0.7	222.0 ± 21.0	8.9 ± 1.1	5.3 ± 1.3	18.0 ± 1.0	2.4 ± 0.1
Female rats										
NC	25.0 ± 4.0	108.0 ± 13.0	56.5 ± 1.6	34.2 ± 1.4	2.5 ± 0.2	121.0 ± 23.0	8.0 ± 1.7	5.5 ± 0.6	21.0 ± 2.0	2.5 ± 0.0
LD	28.0 ± 5.0	123.0 ± 15.0	56.7 ± 1.2	34.0 ± 1.1	2.5 ± 0.2	121.0 ± 31.0	6.8 ± 1.4	5.7 ± 0.09	23.0 ± 3.0	2.5 ± 0.0
MD	22.0 ± 2.0	110.0 ± 18.0	56.2 ± 3.2	33.8 ± 2.1	2.4 ± 0.4	135.0 ± 35.0	8.5 ± 0.6	5.4 ± 1.8	22.0 ± 4.0	2.5 ± 0.1
HD	23.0 ± 4.0	96.0 ± 15.0	57.3 ± 1.5	34.5 ± 1.2	2.0 ± 0.5	115.0 ± 27.0	9.2 ± 0.7	5.4 ± 0.9	22.0 ± 1.0	2.5 ± 0.1

**Groups**	**P** **(mmol/L)**	**TCHO** **(mmol/L)**	**TG** **(mmol/L)**	**K** **(mmol/L)**	**Na** **(mmol/L)**	**Cl** **(mmol/L)**	**GLB** **(g/L)**	**ALB/GLB**	**CK** **(U/L)**

Male rats									
NC	2.8 ± 0.1	1.4 ± 0.2	0.29 ± 0.06	4.9 ± 0.3	142.0 ± 2.0	104.0 ± 2.0	21.5 ± 0.5	1.5 ± 0.0	407.0 ± 103.0
LD	3.1 ± 0.2	1.4 ± 0.1	0.28 ± 0.03	5.0 ± 0.1	141.0 ± 1.0	104.0 ± 1.0	22.0 ± 0.7	1.4 ± 0.1	369.0 ± 83.0
MD	3.0 ± 0.6	1.5 ± 0.3	0.33 ± 0.11	5.0 ± 0.2	142.0 ± 1.0	103.0 ± 1.0	22.7 ± 1.2	1.4 ± 0.1	386.0 ± 160.0
HD	2.9 ± 0.3	1.5 ± 0.2	0.36 ± 0.02	4.9 ± 0.3	140.0 ± 2.0	103.0 ± 2.0	21.7 ± 1.5	1.5 ± 0.1	348.0 ± 84.0
Female rats									
NC	2.8 ± 0.2	1.3 ± 0.4	0.23 ± 0.02	4.4 ± 0.3	141.0 ± 1.0	105.0 ± 2.0	22.4 ± 0.8	1.5 ± 0.1	353.0 ± 94.0
LD	2.8 ± 0.2	1.5 ± 0.2	0.29 ± 0.05	4.4 ± 0.3	141.0 ± 1.0	104.0 ± 1.0	22.7 ± 1.0	1.5 ± 0.1	446.0 ± 153.0
MD	2.7 ± 0.1	1.4 ± 0.3	0.25 ± 0.04	4.6 ± 0.4	141.0 ± 1.0	104.0 ± 1.0	22.4 ± 1.5	1.5 ± 0.1	343.0 ± 83.0
HD	2.4 ± 0.1*	1.4 ± 0.4	0.33 ± 0.07*	4.6 ± 0.3	140.0 ± 1.0	103.0 ± 0.0	22.8 ± 0.7	1.5 ± 0.1	280.0 ± 88.0

ALT, alanine aminotransferase; AST, aspartate aminotransferase; TP, total protein; ALB, albumin; TBIL, total bilirubin; ALP, alkaline phosphatase; GGT, γ-glutamic acid aminotransferase; sGLU, serum glucose; CRE, creatinine; TCHO, total cholesterol; TG, triglyceride; GLB, globulin; CK, creatine kinase; Ca, calcium; P, phosphorus; K, potassium; Na, sodium, Cl, chlorine.

“*” refers to a significant difference from the NC group at *p* ≤ 0.05.

**TABLE 4:** Effects of aqueous extracts of *Cyclocarya paliurus* leaves on the urinalysis parameters of Sprague-Dawley (SD) rats in the acute toxicity study.

Groups	Males			Females		
		
	pH	SG	Volume (ml)	pH	SG	Volume (ml)
NC	6.5 ± 0.0	1.02 ± 0.01	14.0 ± 13.0	6.5 ± 0. 0	1.02 ± 0.00	11.0 ± 4.0
LD	6.7 ± 0.3	1.01 ± 0.00	22.0 ± 11.0	6.9 ± 0.2	1.01 ± 0.01	17.0 ± 11.0
MD	6.7 ± 0.3	1.01 ± 0.00	21.0 ± 8.0	6.6 ± 0.2	1.03 ± 0.01	6.0 ± 3.0
HD	6.7 ± 0.3	1.02 ± 0.00	14.0 ± 5.0	6.7 ± 0.3	1.03 ± 0.01	8.0 ± 3.0

SG refers to specific gravity.

To investigate the effects of AECPL on serum biochemistry, the contents of alanine aminotransferase (ALT), aspartate aminotransferase (AST), total protein (TP), albumin (ALB), total bilirubin (TBIL), alkaline phosphatase (ALP), γ-glutamic acid aminotransferase (GGT), serum glucose (sGLU), urea, creatinine (CRE), total cholesterol (TCHO), triglyceride (TG), globulin (GLB), creatine kinase (CK), calcium (Ca), phosphorus (P), potassium (K), sodium (Na), and chlorine (Cl) in serum samples were measured. The results, presented in Table 3, showed that no significant differences (*p* ≥ 0.05) were found in male rats within all the intragastric doses, compared with the NC group. A similar phenomenon was observed in female rats, expect for the contents of TG and P, which were significantly different (*p* < 0.05) from the NC group when administered with a high dose of AECPL (5,000 mg/kg BW/day) for 14 days.

The effects of AECPL on the urinalysis parameters were evaluated as well. The color and clarity of the urine were yellow or light yellow and clear, respectively, in all the tested rats. As displayed in Table 4, there were no significant (*p* ≥ 0.05) differences in the pH value, specific gravity (SG), and volume between the AECPL-treated groups and the NC group. Parameters such as epithelium, casts, mucus, sperm, trichomonad, RBC, WBC, glucose, bilirubin, occult blood, and urobilinogen were negative in the urine of all the tested animals. The levels of other parameters, which included crystal, bacterium, yeast, ketones, and protein in the AECPL-treated groups were similar to the NC group, and no significant differences found.

Clinical chemistry and hematological parameters have been considered to be important indicators in determining toxicity (22). The decrease of RET in females of the LD group might be an indicator of anemia (23). However, the number of RBC, HGB, WBC, and PLT in females of the LD group was normal, suggesting that the significant changes might be due to biological variation. The absolute value of EOS in females of the LD group was significantly higher than that of the NC group, whereas there was no significant difference in the percent of EOS, implying that no adverse effect of AECPL on EOS of SD rats. A significant increase in TG was observed in high-dose-treated females. However, a 13-week oral gavage toxicity study conducted by our company showed that a TG-lowering effect was induced by a higher dose (10,000 mg/kg BW) AECPL treatment (0.44 mmol/L), in comparison with vehicle treatment (0.53 mmol/L) in females. Similar to a previous *in vivo* study, *C. paliurus* leaves extract could reduce TG content in hyperlipidemic mice (4). All these results suggest that the higher TG content induced by AECPL treatment in HD group females might be due to biological variation. In addition to TG, other parameters like TCHO related to lipids profile were not affected by AECPL treatment. ALT, AST, TP, ALB, GLB, TBIL, ALP, and GGT are important parameters related to liver prolife (24). And no significant differences were observed among the tested groups. All these results indicated that no side effects on liver function were induced by AECPL in the repeated oral dose toxicity study.

### Effects of aqueous extracts of *Cyclocarya paliurus* leaves on the organ weights of Sprague-Dawley rats in repeated oral dose toxicity study

The gross necropsy included examination of the carcass and musculoskeletal system, all external surfaces and orifices, cranial cavity, and external surface of the brain, and contents of the thoracic, abdominal, and pelvic cavities. There were no gross lesions visible in all the tested rats. Additionally, the organs or tissues were weighted. The final results were expressed as the ratio of organ or tissue weight to BW. As shown in Table 5, the values of AG/BW, brain/BW, heart/BW, kidneys/BW, liver/BW, testes/BW, spleen/BW, and thymus/BW in AECPL-treated male rats were not significantly different from that of vehicle treatment males. Similarly, no significant difference was observed for these values except for the ratio of liver/BW in high-dose-treated female rats, in comparison with the females from the NC group. All these results indicated that treatment with AECPL did not cause any abnormalities for the organs like AG, brain, heart, kidneys, liver, testes, spleen, and thymus. However, by comparing with the vehicle-treated females, significantly higher relative liver weight was found in high-dose AECPL-treated females. Previous reports indicated that polysaccharides and/or flavonoids showed protective effects against liver injury *in vitro* (25, 26), suggesting that AECPL might possess health benefits for the liver. The effects of AECPL on the liver need further investigation like pathology analysis.

**TABLE 5:** Effects of aqueous extracts of *Cyclocarya paliurus* leaves on the organ weights of Sprague-Dawley (SD) rats in the acute toxicity study.

Groups	AG/BW (10^–3^)	Brain/BW (10^–3^)	Heart/BW (10^–3^)	Kidneys/BW (10^–3^)	Liver/BW (10^–3^)	Spleen/BW (10^–3^)	Testes (Ovary)/BW^#^ (10^–3^)	Thymus/BW (10^–3^)
Male rats								
NC	0.21 ± 0.04	7.10 ± 0.40	3.74 ± 0.16	8.80 ± 0.30	31.00 ± 1.60	2.35 ± 0.15	9.86 ± 0.44	2.04 ± 0.19
LD	0.17 ± 0.04	7.10 ± 0.30	3.78 ± 0.33	9.30 ± 0.30	31.40 ± 1.50	2.44 ± 0.50	10.60 ± 1.34	2.51 ± 0.17
MD	0.18 ± 0.04	7.10 ± 0.50	3.97 ± 0.24	9.10 ± 0.70	32.30 ± 1.80	2.22 ± 0.41	10.09 ± 0.48	2.15 ± 0.56
HD	0.19 ± 0.02	7.30 ± 0.80	3.83 ± 0.35	9.50 ± 0.50	34.40 ± 3.40	2.48 ± 0.45	10.88 ± 1.38	1.94 ± 0.30
Female rats								
NC	0.33 ± 0.04	9.80 ± 0.60	4.05 ± 0.30	8.80 ± 0.90	30.00 ± 1.10	2.56 ± 0.33	0.47 ± 0.08	3.05 ± 0.38
LD	0.30 ± 0.03	9.80 ± 0.50	4.20 ± 0.26	8.60 ± 0.60	30.10 ± 2.10	2.51 ± 0.31	0.48 ± 0.05	2.65 ± 0.63
MD	0.35 ± 0.06	9.90 ± 0.60	3.89 ± 0.18	9.30 ± 0.60	33.00 ± 2.60	2.62 ± 0.22	0.49 ± 0.03	2.65 ± 0.57
HD	0.33 ± 0.04	9.50 ± 0.60	4.11 ± 0.47	8.60 ± 0.60	34.50 ± 2.10**	2.45 ± 0.28	0.46 ± 0.07	2.76 ± 0.14

^#^Indicates testes for males, ovary for females.

**Refers to a significant difference from the NC group at *p* ≤ 0.01.

### Effects of aqueous extracts of *Cyclocarya paliurus* leaves on the histopathology of Sprague-Dawley rats in repeated oral dose toxicity study

**FIGURE 4:**
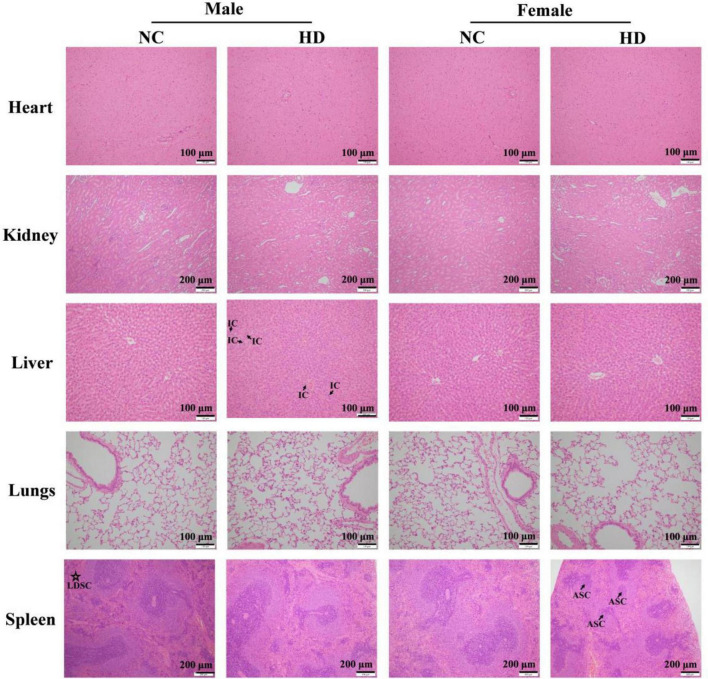
Photomicrograph of heart, kidney, liver, lungs, and spleen of NC and HD groups after H&E staining in acute toxicity study. The photomicrographs of the liver in a male rat of HD group, spleen in a male rat of NC group, and spleen in a female rat of HD group were chosen as abnormities were observed, and other photomicrographs were selected randomly. IC, LDSC, and ASC refer to inflammatory cells, lower density of spleen cells, and atrophied spleen corpuscles, respectively.

The organs such as heart, kidney, liver, lungs, and spleen for histopathological evaluation from the NC and HD groups were processed with hematoxylin and eosin (H&E) staining and microscopic examination (15). The results are shown in Figure 4. As for the liver in high dose-treated females, no visible lesion and inflammatory cell infiltration was observed, similar to female rats in the NC group. All these results implied that the increase in liver weight induced by high-dose AECPL in female rats might be due to biological variation as no significant changes were observed for histopathology. However, slight inflammatory cell infiltration was visible in one of five high-dose-treated male rats, which might result from biological variation as no other abnormities were observed. Additionally, no visible lesion, inflammatory cell infiltration, and necrosis were observed for the organs such as the heart, kidney, and lungs in all the tested animals. However, in the case of the spleen, a lower density of spleen cells in the red pulp was visible in one of the five male rats in the NC group, when compared with those of males in the HD group. While comparing with the females in the NC group, some spleen corpuscles were atrophied or disappeared in the spleen of one of the five females of the HD group, as well as the decreased area ratio of white pulp to a red pulp. However, all the structures of spleen cells were intact without any obvious rupture, suggesting no significant effects on the spleen of tested rats. Additionally, polysaccharides from *C. paliurus* leaves showed a positive effect on mouse spleen lymphocytes, indicating immunomodulatory activities of *C. paliurus* leaves extract (27). All these results indicated that no adverse effects were observed in high-dose-administered SD rats, suggesting that the non-observed-adverse-effect level (NOAEL) of AECPL was 5,000 mg/kg BW/day, the highest level tested in SD rats. However, the effects of AECPL on sub-chronic toxicity were not included in this work, and further study will be conducted in our future work.

## Conclusion

In summary, AECPL was rich in phenolics, flavonoids, and polysaccharides, which have been proven to possess various bioactivities which were responsible for the health benefits of *C*. *paliurus* leaves. The MTD of AECPL was considered to be more than 10,000 mg/kg in SD rats, including male and female rats. After administration once daily for 14 days by oral gavage at dosages of 1,000, 2,000, or 5,000 mg/kg BW/day, the rats were well-tolerated and did not result in any treatment-related mortality and any adverse effects in clinical signs. Moreover, AECPL showed no significant effects on the body weights, food consumption, hematology, coagulation, serum chemistry, urinalysis parameters, organ weights, gross finding, and histopathology. Based on the above results, the NOAEL for AECPL in the present work was considered to be 5,000 mg/kg BW/day for the tested rats.

## Data availability statement

The original contributions presented in this study are included in the article/supplementary material, further inquiries can be directed to the corresponding author.

## Ethics statement

This animal study was reviewed and approved by WuXi AppTec Institutional Animal Care and Use Committee. Written informed consent was obtained from the owners for the participation of their animals in this study.

## Author contributions

CL: investigation, data curation, and writing – original draft preparation. MZ and LW: investigation and data curation, reviewing, and editing. HZ: conceptualization, methodology, and supervision. All authors contributed to the article and approved the submitted version.
